# Caveat emptor: predicting and modeling protein–DNA recognition and binding via machine-learning computational approaches

**DOI:** 10.1093/nar/gkag608

**Published:** 2026-06-25

**Authors:** Morgan A Esler, Rachel Werther, Lindsey A Doyle, Natalia C Ubilla-Rodriguez, Jeanette S Schwensen, Jazmine P Hallinan, Abigail R Lambert, Juliana C Young, Miriam Silverstein, Barry L Stoddard

**Affiliations:** Division of Basic Sciences, Fred Hutchinson Cancer Center, 1100 Fairview Ave. N., Seattle, WA 98109, United States; Division of Basic Sciences, Fred Hutchinson Cancer Center, 1100 Fairview Ave. N., Seattle, WA 98109, United States; Division of Basic Sciences, Fred Hutchinson Cancer Center, 1100 Fairview Ave. N., Seattle, WA 98109, United States; Division of Basic Sciences, Fred Hutchinson Cancer Center, 1100 Fairview Ave. N., Seattle, WA 98109, United States; Division of Basic Sciences, Fred Hutchinson Cancer Center, 1100 Fairview Ave. N., Seattle, WA 98109, United States; Division of Basic Sciences, Fred Hutchinson Cancer Center, 1100 Fairview Ave. N., Seattle, WA 98109, United States; Division of Basic Sciences, Fred Hutchinson Cancer Center, 1100 Fairview Ave. N., Seattle, WA 98109, United States; Division of Basic Sciences, Fred Hutchinson Cancer Center, 1100 Fairview Ave. N., Seattle, WA 98109, United States; Division of Basic Sciences, Fred Hutchinson Cancer Center, 1100 Fairview Ave. N., Seattle, WA 98109, United States; Division of Basic Sciences, Fred Hutchinson Cancer Center, 1100 Fairview Ave. N., Seattle, WA 98109, United States

## Abstract

The recent development of AI-based predictive tools, such as AlphaFold3, for the prediction of the structures of biological molecules and their complexes has transformed modern molecular and cellular biology. While it displays exceptional accuracy in the modeling of folded protein domains and subunits, as well as larger protein–protein complexes and assemblages, AlphaFold3’s performance in predicting the details of protein–DNA (or more broadly, protein–nucleic acid) contacts and complexes is less well established. Here we summarize the recent development and performance of tools intended to predict, model, and/or design protein:DNA recognition and contacts, and then demonstrate (using a well-defined system that offers a minimal “degree of difficulty”) the issues that often surround the use of a resource such as AlphaFold3 for predicting protein:DNA interactions. Beyond providing a cautionary tale for casual users, we note that the incorporation of hybrid models of protein–DNA complexes (in which computationally predicted models are docked into low-resolution CryoEM density maps with little further refinement or quality control) into future training sets may lead to an ongoing and inappropriate learning cycle that further encourages such tools to generate new, equally inaccurate models of protein–DNA complexes.

## Graphical Abstract

**Graphical Abstract:**
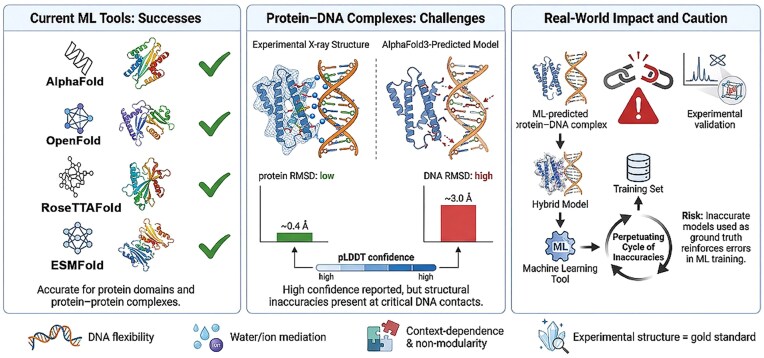

## Introduction

Recently developed computational tools, including machine-learning platforms and algorithms such as AlphaFold3 [1], RoseTTAFold [2], ESMFold [3], and related open-source software platforms such as OpenFold3 (recently reviewed collectively in [4]), excel at accurately predicting and modeling protein folds and protein complexes [5]. Such tools, which leverage the enormous information content that can be extracted from hundreds of thousands of experimentally determined protein structures and many millions of related protein sequences, have significantly impacted a wide range of molecular biology experiments and research projects. Recent examples of the growing influence of such predictive algorithms include a description of the evolution and breadth of the eukaryote-specific viral proteome [6], a broad analysis of the structural and functional effects of alternative splicing on human proteins [7], and the execution of virtual protein–protein pull-down experiments [8]—the output of which could then be rapidly validated on a case-by-case basis [9]. As well, such computational models have greatly facilitated the interpretation of CryoEM maps of large protein assemblages that would otherwise prove exceedingly challenging to accurately model [10].

Despite such advances, the prediction and modeling of DNA-binding specificity [11], the identification of potential DNA-binding proteins from their sequence [12], and/or the design of DNA-binding proteins with desired specificities [13] all remain a significant challenge [12, 14, 15]. The difficulties that are inherent in this area are illustrated by a recent study that described the computational design of sequence-specific DNA-binding proteins via the application of computational machine-learning tools augmented with atomistic physics-based energy approaches [16]. That study, which generated designs constructed upon the small, well understood “helix-turn-helix” DNA-binding motif, required the synthesis and screening of hundreds of thousands of designs, and ultimately produced high-affinity binders against their desired targets for <1 in 10^5^ of those constructs. Many of those rare “hits” were subsequently shown to display degenerate or unintended DNA recognition specificity.

A more recent design effort has described a generative diffusion network approach (termed “RFDpoly”) that allows for enhanced sampling of nucleic acid conformations [17]. While that approach demonstrates improved overall performance, with multiple high-affinity DNA-binding proteins being generated that associate with their intended targets in a sequence-specific manner, the number of designs that display intended DNA recognition and binding activity that rivals naturally occurring DNA-binding proteins still appears to be quite low.

The sub-optimal accuracy and reliability of such computational tools, when applied to the problem of predicting the structural features surrounding protein–nucleic acid binding recognition, appears to be caused by several factors:

1. Intrinsic DNA conformational flexibility, along with protein-induced DNA bending, is a critical feature of sequence-specific DNA recognition [18]. However, the dynamic behavior and “bendability” of DNA (including significant distortion of Watson–Crick basepairs and basepair steps, formation of alternative basepair interactions, and larger-scale deformations of DNA backbone conformation) are extremely difficult to sample adequately via current computational approaches.
2. Solvent molecules and counterions are also highly variable (and extremely important) participants in the formation of protein–DNA contacts, as well as in modulating DNA dynamic flexibility and conformational preferences [19]. However, their presence and participation in protein–DNA interfaces are also very challenging to systematically and accurately sample and model during computational prediction and design efforts.
3. Directional hydrogen bonds and electrostatic interactions play important roles in establishing the requisite balance of affinity versus specificity during protein–DNA recognition and binding [20, 21]. However, the energetics of such interactions are highly dependent upon small changes in interatomic geometries and are also challenging to compute accurately. While this point is also applicable for protein–protein recognition and binding, those interfaces are largely populated with a significantly higher percentage of hydrophobic residues and corresponding non-polar van der Waals contacts [22].
4. Finally (and with only very rare exceptions such as TAL effectors [23]), protein:DNA recognition is a distinctly non-modular, context-dependent phenomena [24–26]. A single basepair substitution within a given DNA target site and/or individual substitutions in a DNA-contacting residue within the protein can lead to significant alterations in the packing and conformation of neighboring DNA nucleotides and protein side chains and in corresponding protein–DNA contacts. This behavior, in which small changes at a basepair position in a DNA target and its most immediate protein contact have an unpredictable but significant effect on the contacts and structural features at neighboring positions in the protein–DNA interface, has been extensively studied and described using a wide range of sequence-specific systems, including Cre-type recombinases [27], homing endonucleases [28, 29], and many different types of transcription factors. A particularly informative example of the latter type of DNA binding proteins’ recognition behavior can be found in analyses of the SOX/TCF (SRY-related high-mobility group box/T-cell factor) family of transcriptional regulators, which illustrated the significant interdependence and “cross-talk” between neighboring base pairs and corresponding protein side chains within defined regions of such proteins’ DNA binding sites [30].

The challenge imposed by the sheer magnitude and number of structural variables and corresponding degrees of freedom described earlier is compounded by the availability of far fewer experimentally determined protein–DNA complex structures as compared to the wealth of structures of purely protein-based folds and assemblages [31]. That disparity is further exacerbated by the fewer number of correlated sequences of homologous DNA-binding proteins and their corresponding DNA targets that can be confidently aligned and thereby further inform investigators and algorithms about the co-evolution of protein–DNA binding partners (e.g. see [32]), as compared to the wealth of similar information available for analyzing the co-evolution of protein–protein complexes [33]).

In an attempt to improve protein–DNA complex prediction accuracy, a variety of studies have attempted to employ “hybrid” approaches that integrate atomistic, physics-based energy calculations and modeling with machine learning. One such effort, leading to the development of a tool termed “DDPScore,” corresponds to a 4D convolutional neural network trained on DNA–protein decoy structures generated from physics-based docking methods; it is claimed to outperform traditional scoring functions, especially in flexible docking scenarios [34]. In a subsequent complementary approach, investigators combined molecular dynamics simulations and MM-GBSA energy calculations with machine-learning models—including neural networks and support vector machines—to predict transcription factor–DNA binding affinities. Their model achieved high correlation with experimental binding data and provided interpretable energetic features that revealed the molecular basis of specificity [35]. Together, these studies demonstrate how physics-derived features and structural ensembles can enhance the accuracy, ranking, and biological insight of AI-based protein–DNA prediction.

Given the state of the field described earlier, it is perhaps concerning that several machine-learning-based tools that have been recently developed to predict protein–DNA contact positions (such as GraphSite [36], EquipNAS [37], and GraphNABP [38]) appear to include computationally modeled protein–DNA complexes—primarily generated using AlphaFold—in addition to experimentally-determined structures to train their algorithms [39].

To anecdotally illustrate, using the simplest possible system, the issues surrounding the reliability of machine-learning tools for the prediction and modeling of protein–DNA complex structures, we have compared a series of recently determined X-ray crystal structures of a series of variants of a well-characterized DNA-binding model protein that display local alterations of DNA recognition specificity against corresponding computational models (generated using AlphaFold3) of the same complexes. The model protein used for this analysis, known as I-OnuI, is a member of a family of DNA-binding proteins known as “LAGLIDADG homing endonucleases” [40], which are also referred to as “meganucleases” in the literature [41].

We chose to use the I-OnuI homing endonuclease for this exercise for several reasons:

1. As much as any other DNA-binding protein, DNA-bound complexes of I-OnuI were well-represented in the structural database that provided the core training set for AlphaFold3 during its development. The wild-type DNA-bound cocrystal structure of I-OnuI was originally deposited and released in 2011 [42] and was followed by multiple related structures of variants that recognize altered DNA targets, all deposited and released in 2017 and 2018 [29].
2. Several additional DNA-bound cocrystal structures of closely related evolutionary homologues of I-OnuI (that display closely superimposable protein and DNA conformations along with moderately diverged protein sequences) were deposited in and released by the PDB from 2011 through 2018 [43] and were also available for training purposes during the training of AlphaFold3.
3. The specificity of I-OnuI can be readily altered at individual basepairs or short runs of two to three sequential basepairs, via a straightforward process of mutagenizing nearby protein residues and then selecting for a protein variant that exhibits binding and cleavage of the altered target site [29, 44, 45].
4. The alteration and divergence of homing endonuclease specificity (regardless of being accomplished during evolution or in the laboratory) is accompanied by minimal alteration of either the protein structure or the overall bending and conformation of the DNA target. Thus, differences in many of the structural features that contribute to indirect readout of the DNA sequence (i.e. binding-induced DNA bending and accompanying protein conformational changes) are minimal when comparing such related DNA-binding proteins bound to their targets.

We note that while LAGLIDADG homing endonucleases clearly adhere to the same principles governing protein–DNA recognition as any other family of DNA-binding proteins, they have evolved to act as mobile genetic elements, including rapidly evolvable target recognition specificity and behavior. Therefore, they may not perfectly mirror the behavior and predictability other DNA-binding protein families that participate in alternative biological functions and pathways and that display different balances of affinity and specificity.

## Fundamental details of DNA conformation, solvation, side-chain rotamers, and protein–DNA contacts for minimally altered variants of I-OnuI are very poorly predicted

**Figure 1.**
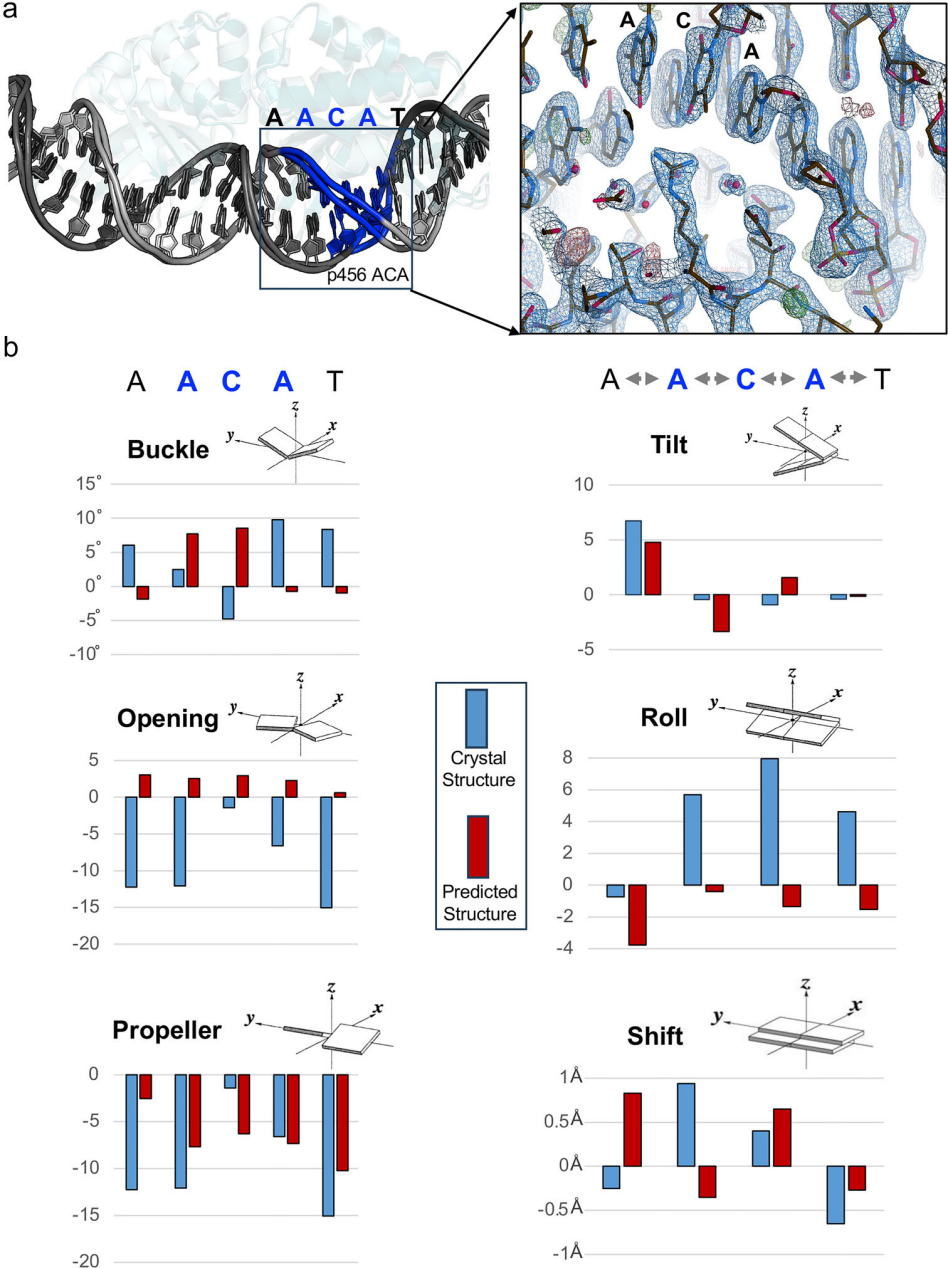
Comparison of the overall protein–DNA complex and DNA conformation for the I-OnuI variant “eI-OnuI_P456_ACA (PDB ID 9XY9) and a corresponding AlphaFold3 (AF3) model of the same complex. Panel a: Superposition of the crystal structure and the AF3 model. The region of the protein and corresponding DNA target that differ from otherwise unchanged wild-type protein and DNA target (termed “P456,” corresponding to “Positions +4,+5, and +6” in the DNA target) is indicated by the box and blue DNA backbone and bases. Note the deformation of DNA backbone within that region, relative to the immediately flanking basepairs of the DNA target. The corresponding electron density and modeled bases and side chains are shown in the boxed inset. Panel b: Comparison of the crystallographically observed and AF3-predicted values for six different DNA conformational parameters, displayed across the P456 “ACA” sequence (as well as the flanking 5′ and 3′ basepairs that immediately precede and follow that sequence). Conformations of three values corresponding to the conformation of individual base pairs (buckle, opening, and propellar twist) are shown in the left graphs; geometries of three values corresponding to individual base pair steps (tilt, roll, and shift) are shown in the right graphs. The values for this figure and for Fig. 2 were generated using the 3DNA Webserver [58], and the illustrations of individual basepair and base step deformations were copied from that same online resource.

To minimize the “degree of difficulty” faced by AlphaFold3 in our initial comparisons of structures and predicted models, 13 individual constructs were created that differ from the wild-type I-OnuI:DNA complex across only a small contiguous region of the protein–DNA interface, surrounded by otherwise unchanged, wild-type protein and DNA structure and sequence (Fig. 1a and Table 1). Each protein construct differs from wild-type I-OnuI at five to eight DNA-contacting residues located in a localized cluster, corresponding to only 2%–3% of the protein sequence; their corresponding DNA targets differ at no >3 consecutive basepairs within the same region, embedded within the protein’s surrounding and otherwise unchanged target. This group of protein residues and DNA basepairs is referred to below as the “Position 4,5,6” or “P456” region, corresponding to the numbering system for the enzyme’s DNA target site.

**Table 1.** Summary of comparison of crystallographic structures to AlphaFold3 (AF3) predictions of “modular” variants of the I-OnuI homing endonuclease bound to variants of that protein’s DNA target site, and of the DNA-bound crystal structure of I-PnoMI to a corresponding AF3 prediction of that complex

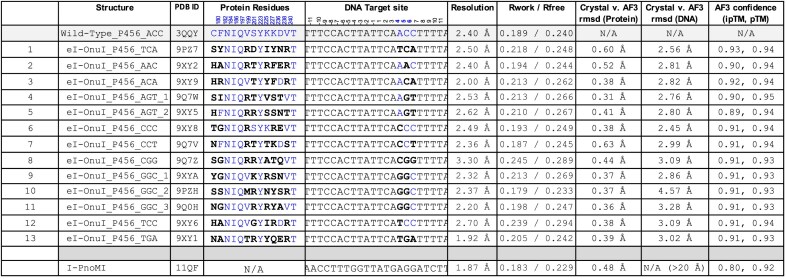

The altered protein residues in the “P456” region and the corresponding three-basepair sequence for each construct are indicated with bold font. The crystal structures, data, validation reports, and maps are also publicly available at the protein structural database (www.rcsb.org) [52]; summaries of all 14 data sets and refinements are further provided in Supplementary Table S1. PDB files of the refined crystal structures (corresponding to the PDB ID codes shown in column 3) and the predicted protein–DNA complexes generated by AF3, along with a Pymol session file with superimposed proteins and the final .mtz file with map coefficients for each structure, are also provided in a downloadable online Supplementary Folder coupled to this article.

The variants of I-OnuI described earlier were generated via a previously described selection approach [44–46] mentioned earlier. A small number of protein residues located in immediate proximity to the corresponding altered DNA bases were subjected to combinatorial mutagenesis, followed by selection for site-specific recognition of the altered DNA target. The structure of each unique I-OnuI variant bound to its corresponding DNA target site was then visualized via X-ray crystallographic analyses (Table 1 and Supplementary Table S1), using methods for purification and crystallization that have been previously described [29, 42] and that are also described in Supplementary Table S1 and further provided in the publicly available PDB entries for each structure. All structures were solved with HKL2000 [47], PHASER [48], Phenix [49], COOT [50], and CCP4 [51], then submitted to the Protein Data Bank (RCSB.org) [52] with the PDB codes shown in Table 1.

Separately, the amino acid sequence of each protein variant and its corresponding DNA target site was submitted to the AlphaFold3 server for prediction of the DNA-bound structure and corresponding protein–DNA contacts. The AF3 model that most closely resembles the crystallographic structure was used as the basis for detailed comparison—a choice that provided the AF3 server and prediction the best opportunity to succeed (in the end, the choice of which AF3 model to focus on for comparison with the corresponding crystal structure made little difference, as the individual AF3 models were consistently almost identical to one another). For each complex, the global confidence indicated by AF3 was quite high (Table 1), ranging from 0.89 to 0.92 (ipTM) and 0.93 to 0.95 (pTM) scores, with relatively high pLDDT (predicted Local Distance Difference Test) values calculated across each protein chain and DNA duplex, including the altered P456 region (illustrated for one such prediction in Supplementary Fig. S1 and described in detail below).

The resolution of the crystallographic structures of the 13 protein–DNA complexes ranged from a high of 1.92 Å to a low of 2.70. Å. The individual proteins are almost entirely unchanged from one another. Unsurprisingly, the corresponding predictions made by AF3 of each protein’s structure and fold are also very consistent with one another and highly accurate: superpositions of just the protein moieties in each complex (i.e. pairwise comparisons of the protein subunits from each of the crystal structures against their counterparts from the corresponding AF3 models) yielded all-atom RMSD values of 0.36 to 0.63 Å (Table 1).

In contrast, the predicted conformations of the DNA targets bound to each protein were far less accurate. All-atom RMSD values for the various protein-associated DNA duplexes (calculated between the same crystal structures and corresponding AF3 models, after superposition of the bound proteins) were much higher, displaying all-atom RMSD values of 2.45 to 4.57 Å (average 3.01 Å) (Table 1). The deviation between the predicted bound conformation of bound DNA targets and their actual position in the DNA-bound cocrystal structures is largely a function of significant alteration of local DNA backbone and basepair geometries across much of the bound DNA, including multiple DNA conformational parameters within the P456 region.

A detailed example of the differences between a predicted protein–DNA complex and its actual structure is provided here for the “eI-OnuI_P456_ACA” construct (Table 1; PDB ID 9XY9). The discrepancies between predicted and observed DNA conformational parameters across the altered region of the complex correspond to significant differences in both basepair geometries and in basepair steps (Fig. 1b), which in turn are coupled to inaccurately predicted DNA backbone conformation and groove dimensions spanning the same basepairs. Perhaps most striking, beyond the differences in predicted versus experimentally visualized DNA backbone positions spanning the resculpted region, was the number of basepair and base-step parameters within that region that displayed inverted values relative to the conformation of an ideal B-form DNA duplex. For example, the buckle, opening, and roll angles for the central C:G basepair of the P456 “ACA” sequence display differences of up to 13° between the crystallographic structure and the AF3 prediction, with all three parameters “reversed” between the predicted versus observed direction of the basepair deformation.

**Figure 2.**
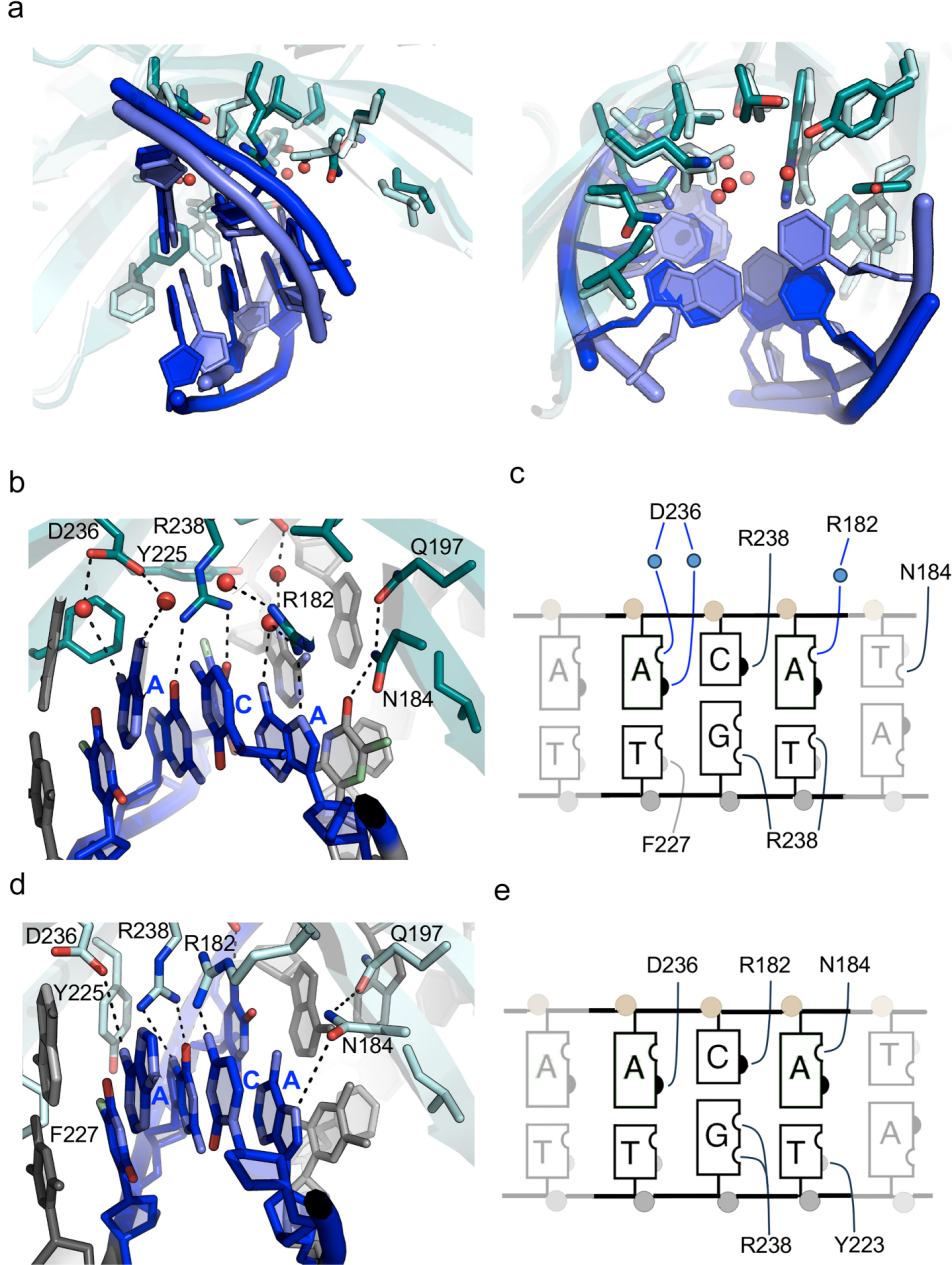
Comparison of the overall protein–DNA complex and protein–DNA contacts for the I-OnuI variant “eI-OnuI_P456_ACA (PDB ID 9XY9) and a corresponding AlphaFold3 model of the same complex within the “P456” region of the protein–DNA complex. The predicted model from AF3 corresponds to that which most closely resembles the crystal structure. Panel a: Superposition of the crystallographic structure and computational model. Panels b and c: Crystallographically observed contacts between protein residues and basepairs in the P456 region of the complex. Three well-ordered water molecules are involved in direct solvent-mediated contacts between protein sidechains and corresponding basepair atoms. Panels d and e: Predicted contacts between protein residues and basepairs in the same P456 region of the complex. The differences in DNA conformation and presence or absence of bound solvent molecules within the interface between the experimental structure and computational model are compounded by dramatic differences in multiple side-chain rotameric conformations, and ultimately by mis-assignment of individual contacts between those side chains and neighboring DNA bases.

The inaccuracies in local DNA conformation noted above are compounded by the prediction algorithm’s inability to assign ordered solvent molecules within the protein–DNA interface (Fig. 2). In the crystal structure of this complex, five such solvent molecules are clearly observed within or immediately adjacent to the DNA basepairs and protein side chains within the P456 region; three of those are directly incorporated into water-mediated direct contacts between individual bases and side chains (Fig. 2b and c). The net result of inaccurate modeling of DNA conformation and corresponding hydration of the protein–DNA interface is significant discrepancy in the assignment of side chain rotameric conformations and corresponding sequence-specific contacts between individual DNA base atoms and protein side chains (illustrated in Fig. 2b and c versus Fig. 2d and e).

Examination of the range and distribution of AlphaFold3 pLDDT values for the predicted structure (Supplementary Fig. S1) does not indicate obvious low confidence in the model (either globally or in the altered P456 region) that would immediately alert a casual user of the tool of the types of inaccuracies described earlier. In particular, the tool displays the most significant reduction in pLDDT values across the protein at regions respectively corresponding to the N- and C-terminal residues and a central, poorly ordered linker connecting the protein’s two folded domains; similarly, the pLDDT values calculated across the DNA target display a sharp minima across the middle four base pairs of the DNA sequence (that corresponds to the maximum bend in the center of the target that LAGLIDADG enzymes consistently induce upon binding) rather than at the P456 basepair positions.

The additional 12 similar exercises using alternative P456 DNA base pairs and corresponding I-OnuI variants display similar degrees of inaccurate modeling of DNA conformation and incorrect assignment of direct protein:DNA contacts. The PDB files of all superimposed crystallographic structures and AF3 models, along with PyMol session files of the same superpositions and final refined .mtz map files for the crystal structures, are provided in Supplementary Data Folder associated with this manuscript. The deposited crystal structures for each complex are also now publicly available at the protein structural database (the RCSB [52]; structure ID codes are provided in Table 1 and Supplementary Table S1).

## The prediction inaccuracies noted above can lead to far more profound issues when addressing a more realistic homology modeling problem of a protein–DNA complex

The model system described earlier, in which only a small region of an otherwise fixed, previously deposited protein and DNA molecular sequence and structure was altered, is obviously an artificial construct intended to reduce the challenge of predicting protein–DNA contacts and recognition to the simplest possible level—and which illustrated significant issues that surround computational attempts to accurately sample and model the vast stereochemical space that defines nucleic acids and the DNA component of any protein–DNA complex.

To examine the extent to which the shortcomings described earlier would impact a more realistic modeling challenge, we extended our analysis to the prediction of the DNA-bound structure of a naturally occurring homing endonuclease named I-PnoMI. That protein displays significant homology both to I-OnuI (the model enzyme used in the prior modeling exercise) and to a related protein named I-SmaMI (Supplementary Fig. S2). I-PnoMI displays 45% sequence identity to the latter protein, along with a similar degree of similarity between their DNA target sites (matched at 10 out of 22 base pairs, also 45% identity). With eight separate high-resolution crystal structures of I-SmaMI bound to DNA available in the protein structural database (all deposited over 10 years ago) [43, 53], modeling an I-PnoMI:DNA complex should represent a straightforward problem in homology modeling that any well-trained molecular biologist might accurately address accurately using a variety of available tools, combined with common sense. For the analysis described below, a small number of point mutations (all distant from the DNA-contacting surface) that improve the protein’s solution behavior were incorporated into its sequence.

**Figure 3.**
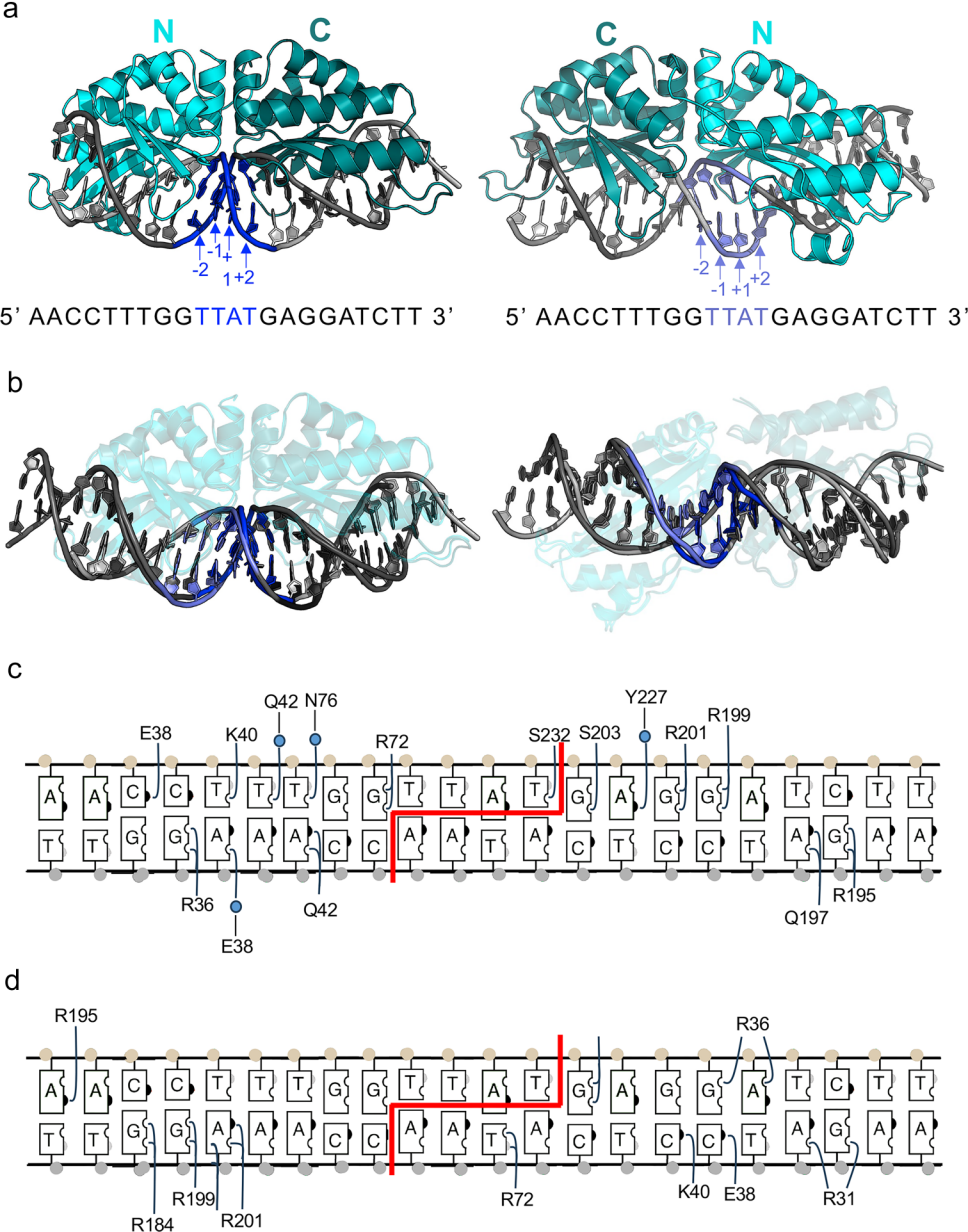
Comparison of the crystal structure of I-PnoMI bound to its DNA target site to a corresponding AlphaFold3 prediction of the same complex. The alignment of I-PnoMI and its target site with its two closest homologues in the protein structure database are shown in Supplementary Fig. S2. Panel a: The crystal structure of I-PnoMI (left) demonstrates the expected alignment and orientation of the enzyme’s N- and C-terminal domains over the 5′ and 3′ DNA half-sites, and enzyme’s active site over the center of the target site. The base-pair positions from −2 to +2 (5′-TTAT-3′ and its complement) correspond to the four-base-pair overhang generated by the enzyme’s cleavage activity. In contrast, the predicted AF3 model (right) places the enzyme in an inverted orientation on the DNA target site, with the center of the target and the corresponding cleavage sites shifted by two full base pairs to one side of the enzyme’s active site. This solution was produced for 4 out of 5 AF3 predictions; the fifth and final solution placed the enzyme in the correct orientation, but again with DNA positions out of register. Panel b: Superposition of the crystallographic structure and predicted model of the I-PnoMI–DNA complex. The DNA duplex is reversed from its correct orientation in the AF3 model and displays significant deformations in backbone geometry. Panels c and d: Schematic demonstration of contacts observed between individual protein side chains and DNA base pairs for the crystallographic structure and the AF3 model, respectively. The crystal structure clearly demonstrates the formation of multiple “canonical” sequence-specific contacts between several arginine residues (R36, 195, 199, and 201) and corresponding guanine bases (see Fig. 4 for electron density), and similar highly specific bidentate contacts between two glutamine residues (Q42 and Q97) and a pair of corresponding adenine bases. It also illustrates the formation of four water-mediated contacts between additional side chains and DNA bases. In contrast, the AF3 model, in which the DNA is inverted and out of register with the protein, places all side chains at incorrect contact positions within the target site and largely fails to predict similar canonical sequence-specific contacts. The red lines indicate the sites of DNA strand cleavage and corresponding positions of overhanging bases in the cleaved product.

The AlphaFold3 server produced a predicted model of the I-PnoMI:DNA complex that is fundamentally incorrect, as illustrated in Fig. 3. The crystal structure of I-PnoMI (left) bound to its target (solved at 1.87 Å resolution) demonstrates the expected alignment and orientation of the enzyme’s N- and C-terminal domains over the DNA target’s 5′ and 3′ half-sites, and correctly places its active sites over corresponding scissile phosphates (which flank basepair positions −2 and +2, corresponding to the eventual generation of a 4-base, 3′ overhang upon cleavage). In contrast, the predicted AF3 model places the enzyme in an inverted orientation on the DNA target site, with its center and corresponding cleavage sites shifted by two full basepairs to one side of the enzyme’s active site. The predicted model also lacks multiple ordered solvent molecules that contribute to sequence-specific contacts and (not surprisingly) displays significant inaccurate deformations of DNA backbone conformation that are largely attributable to the fact that the DNA sequence runs in the reverse orientation of its actual bound position.

This solution was reproduced for 4 out of 5 AF3 predictions; the fifth and final solution placed the enzyme in the correct orientation but was also fully out of register with the center of the target site.

**Figure 4.**
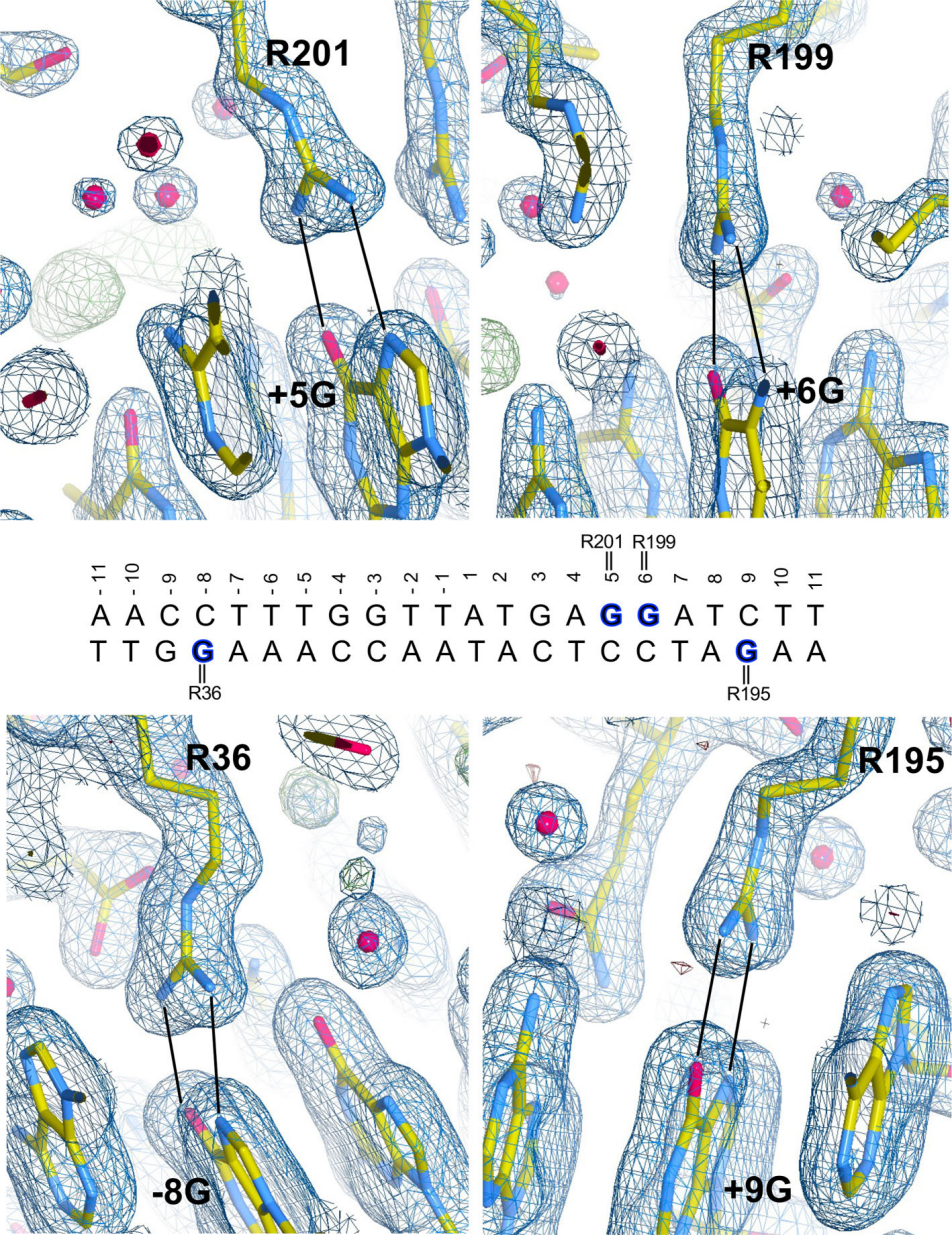
Electron density and corresponding atomic models of the I-PnoMI:DNA complex, displaying the positions and contacts for each of the four sequence-specific Arg:guanine contacts formed in the protein–DNA interface.

In the crystallographic structure of the bound complex, the proper orientation and register of the DNA target places four arginine residues (R36, 195, 199 and 201) and two glutamine residues (Q42 and 197) into sequence-specific, bidentate contacts with corresponding guanine and adenine bases (see Fig. 4 for a view of the electron density corresponding to the arginine-guanine contacts). It is worth noting that these two individual forms of direct readout and contacts between protein side chains and corresponding DNA bases have been well-documented as canonical, sequence-specific DNA contacts for the past 25 years, since their initial cataloguing and description in 2001 (see Fig. 3 in [54]). In addition to failing to produce those highly favorable and well-understood interactions, the computational models instead produce far fewer direct protein–DNA contacts overall; those that are predicted are largely of questionable stereochemical quality.

Where to from here? The anecdotal analysis described above corresponds to one particular, well-defined system that illustrates the gap between the most commonly employed AI-based molecular modeling tool, versus the multiple parameters and corresponding degrees of freedom that actually dictate the formation of sequence-specific protein:DNA complexes. The particular system used in this analysis is, in many ways, as simplified as possible. The protein system being used is quite well-studied and historically well-represented in the protein structural database, and binding of the DNA target involves relatively minimal, and highly consistent, bending of the DNA duplex with no significant departure from canonical Watson-Crick base pair geometries. Obviously, the degree of difficulty in the accurate prediction and modeling of protein:DNA complexes would likely be increased for most, if not all, other types of modeling challenges involving protein–DNA binding.

We note that in contrast to the results described here, there are numerous anecdotal examples of the AlphaFold3 server accurately predicting DNA recognition specificity and corresponding complexes (recently listed and reviewed for example in [55]). Such cases may reflect individual families of DNA binding proteins that are represented in the PDB in sufficient numbers and diversity to produce individual cases that perform well. Additionally, certain structural folds associated with DNA binding may intrinsically lend themselves to being more readily predictable due to unique structural features that are not yet fully appreciated.

As noted in the introduction to this piece, it may be (and indeed seems quite likely unless many new, additional high resolution structures of protein–DNA complexes are added to the structural databases) that more accurate and reliable modeling efforts will require the combination of machine-learning / AI based modeling approaches with parallel or downstream physics- and energy-based modeling and refinement strategies, including Rosetta-based docking and scoring, molecular dynamics refinement of initial models, explicit modeling of bound solvent and corresponding water-medicated protein–DNA contacts, and improved prediction of DNA conformation. A pair of recently published studies [56, 57] have proposed such computational strategies as part of future approaches.

There are three primary concerns in this area of research and investigation that merit consideration moving forward. The first, and most obvious, is that casual users of AI-based predictions of molecular complexes and assemblages should employ considerable care in the interpretation and use of such models, at least when they are focused on protein–DNA or protein–nucleic acid complexes: in contrast to the prediction of purely proteinaceous molecular assemblages, such models clearly have elevated potential to be misleading.

The second concern is the likelihood that future training sets of protein:DNA complexes may become increasingly populated with “hybrid models” that correspond to relatively low resolution CryoEM density maps into which AI-generated models of protein:DNA complexes have been docked but otherwise largely left unchanged from the output of the computational server. It would seem likely that such models, which primarily reflect the product of computational modeling with minimal subsequent refinement or validation, may serve to reinforce, rather than improve, the accuracy that would be required in order for users to be able to confidently employ such predictions for detailed experimental design for the study of protein:DNA recognition. Therefore, it would seem crucial that future efforts to further train and improve upon AI-based modeling algorithms should employ steps to carefully cull training sets in a manner that ensures that they are largely comprised of highly accurate, well-validated molecular structures.

The third and final concern is more philosophical in nature. As mentioned above, the generation of a biochemically and structurally reasonable model of a protein such as I-PnoMI, that harbors considerable sequence identity and remarkably similar DNA recognition specificity to several proteins that have been repeatedly visualized via high resolution crystallographic analyses, should be (and hopefully still is) a relatively straightforward problem for many if not most well-trained, experienced molecular biologists. However, it is easy to imagine that the ease of using a tool such as the AlphaFold3 server might lead to increasing, and somewhat blind reliance on such a resource, at the expense of the type of a straightforward (but more thought-requiring) manual analysis that would likely lead to the generation of a reasonable, common-sense model of protein–DNA interactions and function.

Finally, we note that this analysis, while hopefully offering food for thought (and a cautionary tale) for those who use AlphaFold3 in their research, is by no means intended as a criticism of this or any other tool or its many developers, which (as indicated at the top of this piece) have led to a profound revolution in molecular and structural biology that has facilitated and accelerated the study of increasingly complex molecular systems and assemblages. Rather, we hope that future efforts to computationally address the complexities of nucleic acid dynamics, solvation, and chemistry, both in isolation and in protein-bound complexes, if performed in a rigorous manner, will in due course relegate the observations noted here to a snapshot that described the early history of such tools and their improvement.

## Supplementary Material

gkag608_Supplemental_Files

## Data Availability

All crystallographic coordinates and corresponding sequences of the proteins described in this study have been deposited in the protein database (www.rcsb.org) and are available for immediate downloading and examination, via ID codes listed in Table 1 and Supplementary Table S1 of this manuscript. The PDB files of all superimposed crystallographic structures and AF3 models, along with PyMol session files of the same superpositions and final refined .mtz map files for the crystal structures, are provided in a downloadable Supplementary Data Folder associated with this manuscript.

## Notes

Present address: Department of Biological Chemistry, University of Michigan Medical School, 1150 W. Medical Center Dr., Ann Arbor, MI 48109, United States
