# Supplementary material for: Caveat emptor: predicting and modeling protein–DNA recognition and binding via machine-learning computational approaches
Source: Nucleic Acids Res. 2026 Jun 25;54(12):gkag608. doi: 10.1093/nar/gkag608 (PMC13294676; doi:10.1093/nar/gkag608)
Supplement: gkag608_Supplemental_Files [file gkag608_supplemental_files.zip › Supplementary_Information_Figures_Tables.pdf]

Supplementary Figures, Tables and Captions

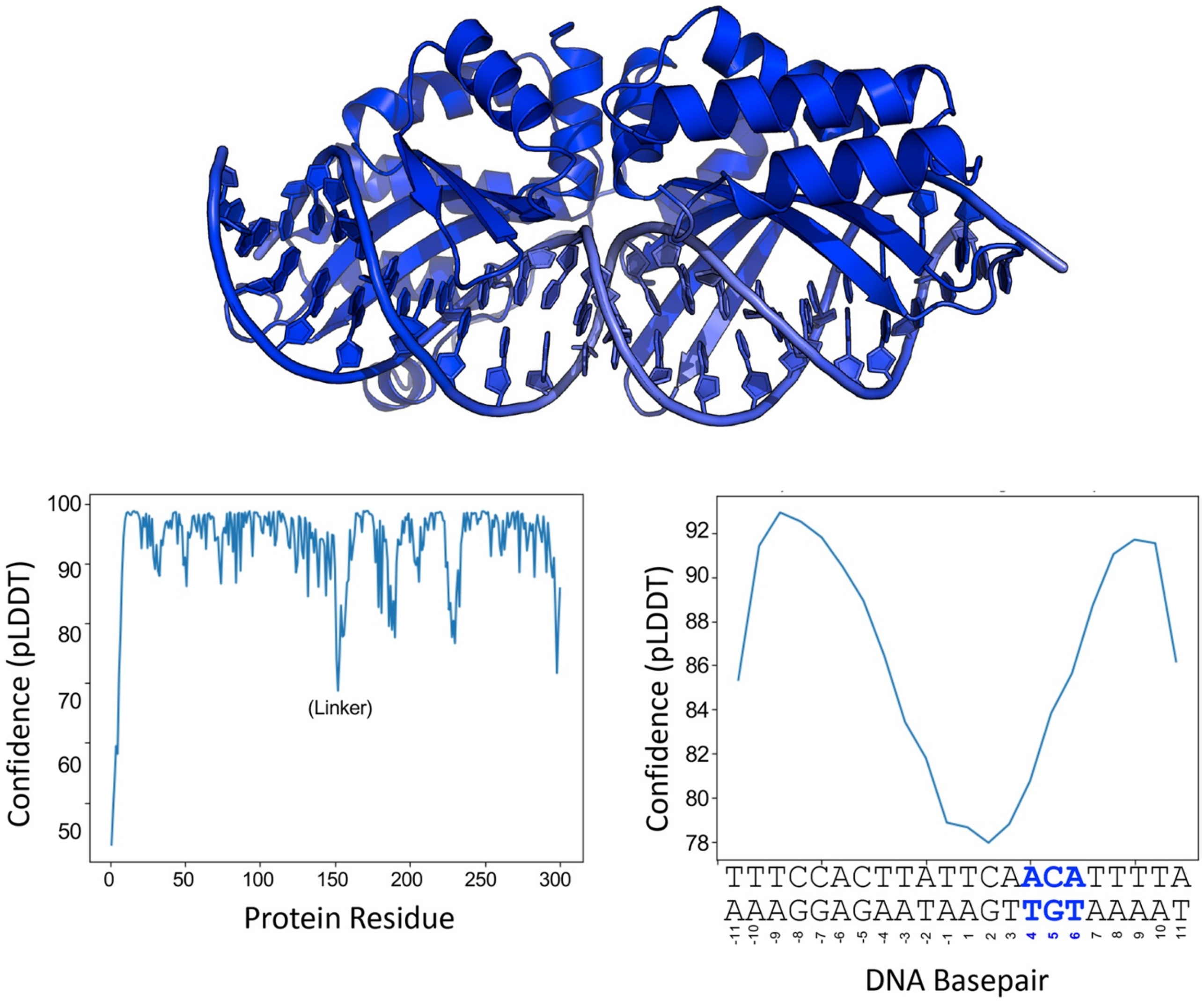

Supplementary Figure S1. Distribution and range of pLDDT (predicted Local Distance Difference Test) scores across the DNA-bound model of eOnu_03_ACA (corresponding to **Figures 1, 2 and 3**). ***Top***: Color-coded distribution for the entire complex; darker blue corresponds to higher pLDDT scores, as typically displayed at the AlphaFold3 server (50). ***Bottom***: Plots of average atomic pLDDT scores for each residue of the protein (left) and each basepairs of the DNA target site. For the latter, the position of the P456 region (corresponding to the 5'-ACA-3' sequence of the top strand) is indicated with bold blue font. Note that the three regions of lowest atomic confidence across the protein chain correspond to the two peptide termini and a poorly ordered linker that connects the two folded domains; the remaining two minima are located near the DNA target, but do not correspond closely to the contact residues in the P456 region as listed in **Table 1**. Also note that the lowest confidence region in the DNA duplex corresponds to the middle four basepairs of the target, where the DNA is most dramatically bent by the protein, rather than the retargeted basepairs of the P456 region.

Protein Sequences:

```
                                                    ↓ ↓ ↓ ↓
I-PnoMI         MYRSTIVNPWVWSGLIDGEGSFSIIISKSKKRKLGWRVELKFQLGLHKKDLNLLELLQQH
I-SmaMI         MGENSKLNPWAVVGFIDAEGSFMVRVRKNSKYKTGWLVVAIFSVTVDKKDLFLLESLKTF
I-OnuI          MSRRESINPWILTGFADAEGSFLLRIRNNNKSSVGYSTELGFQITLHNKDKSILENIQST
                * .   :***   *: *.**** : : :..* . *: .   *.: :.:**  :** ::

                            ↓   ↓
I-PnoMI         LGGIGSIHLAKNRDMVNYSIDSIKDLNNLIDYLDKYPLLTQKAADFLLLKKAVELVNNKA
I-SmaMI         FGGLGSIKKSGNSTFSYRIESSEQLTKIILPFFDKYSLITEKLGDYLLFKKVLELMGTKE
I-OnuI          WKVG--VIANSGDNAVSLKVTRFEDLKVIIDHFEKYPLITQKLGDYMLFKQAFCVMENKE
                      :    .           :  : :: .::**.*:*:* .*::*:*:.. :: .*

I-PnoMI         HLTLEGLEKIVNIKASMNLGLSDMLISEFPGYVPVERPVINNDNVILNPYWISGFVSAEG
I-SmaMI         HLTQRGLEKIVSLKASINKGLSEELQAAFPQCVPTPRPEINNKN-IPDPFWLAGFVSGDG
I-OnuI          HLKINGIKELVRIKAKLNWGLTDELKKAFPEIISKERSLINKN--IPNFKWLAGFTSGEG
                **. .*::::* :**.:* **:: *   **  :.  *. **:.  * :  *::**.*.:*

                                ↓ ↓ ↓ ↓ ↓                       ↓      ↓
I-PnoMI         NFDVRVP-STNSKLGYRVQLRFRISQHSRDLILMQKIVEYLGCGKIYKYAGK--SSISLT
I-SmaMI         SFKSILKKSESIKVGFQSILVFQITQHARDVKLMESLISYLGCGFIEKDSRG--PWLYYT
I-OnuI          CFFVNLIKSKSK-LGVQVQLVFSITQHIKDKNLMNSLITYLGCGYIKEKNKSEFSWLDFV
                 *   :  * .  :* :  * * *:** :*  **:.:: ***** * :      . :  .

I-PnoMI         IVDFKDITNILVPFFDEYPIIGIKLHDYLDWCKIHSLMLNKSHLTVEGINSIRKIKSGMN
I-SmaMI         VTNFSDIQGKIIPFFHQYKIIGSKYGDYMDWCKIALIMQNKNHLTPEGLNEIRALKGGMN
I-OnuI          VTKFSDINDKIIPVFQENTLIGVKLEDFEDWCKVAKLIEEKKHLTESGLDEIKKIKLNMN
                :..*.** . ::*.*.:  :** *  *: ****:  :: :*.*** .*::.*: :* .**

I-PnoMI         TGRNF
I-SmaMI         KGRL-        45% identity (137/303)
I-OnuI          KGRVF        39% identity (117/303)
                .**
```

DNA Target Sequences:

```
I-PnoMI         5′ AACCTTTGGTTATGAGGATCTT 3′
I-SmaMI         5′ TATCCTCCATTATCAGGTGTAC 3′     45% identity (10/22)
I-OnuI          5′ TTTCCACTTATTCAACCTTTTA 3′     23% identity (5/22)
```

Supplementary Figure S2. Alignment of protein sequences and corresponding DNA target sites of I-PnoMI (the subject of a comparative analysis of the DNA-bound crystal structure and corresponding AF3 prediction illustrated in **Figures 4 and 5**) with its evolutionarily related homologues I-SmaI and I-OnuI. I-SmaMI shares 45% identity with I-PnoMI in both its amino acid sequence and across its DNA target site. Arrows indicate the positions of direct contacts between side chains and bases in the I-PnoMI-DNA complex. The crystallographic structure of I-OnuI was deposited in the protein structural database in 2011 (PDB code 3QQY) with six additional structures of I-OnuI variants were deposited and made publicly available in 2017 (PDB ID codes 5T2H, 5T2N, 5T2O, 5T8D, 5THG, and 5V0Q). The first crystallographic structure of I-SmaMI bound to DNA was deposited and made publicly available in 2014 (PDB ID code 4LOX) with seven additional structures deposited in 2016 (PDB ID codes 5E5O, 5E5P, 5E5S, 5E63, 5E67, 4Z1Z, and 4Z20).

PDB ID	9PZ7	9XY2	9XY9	9Q7W	9XY5	9XY8	9Q7V
	P456_TCA	P456_AAC	P456_ACA	P456_AGT_1	P456_AGT_2	P456_CCC	P456_CCT
**Data Collection**							
Space group	P 21 21 21	P 21 21 21	P 21 21 21	P 21 21 21	P 21 21 21	P 21 21 21	P 21 21 21
Cell dimensions							
a, b, c (Å)	49.21, 63.74, 166.75	39.31, 76.16, 167.82	39.35, 76.61, 167.49	39.22, 70.49, 166.86	39.17, 68.38, 165.54	39.22, 76.16, 167.75	49.01, 63.26, 167.05
α, β, γ (°)	90.0, 90.0, 90.0	90.0, 90.0, 90.0	90.0, 90.0, 90.0	90.0, 90.0, 90.0	90.0, 90.0, 90.0	90.0, 90.0, 90.0	90.0, 90.0, 90.0
Resolution (Å)	50.0-2.50 (2.59-2.50)	50.0-2.4 (2.49-2.40)	50.0-2.0 (2.07-2.00)	50.0-2.53 (2.62-2.53)	50.0-2.62 (2.71-2.62)	50.0-2.49 (2.58-2.49)	50.0-2.36 (2.44-2.36)
R_{Merge}	0.13 (0.71)	0.08 (0.32)	0.07 (0.72)	0.10 (0.36)	0.09 (0.66)	0.30 (0.07)	0.09 (0.32)
I/σ(I)	18.1 (2.3)	24.1 (5.0)	35.0 (3.6)	20.8 (3.7)	15.6 (2.0)	24.7 (4.5)	23.1 (4.2)
Completeness (%)	99.5 (97.5)	98.2 (92.3)	99.1 (98.4)	98.6 (95.5)	97.0 (97.6)	99.5 (97.1)	99.4 (95.8)
Redundancy	11.4 (7.7)	6.7 (5.2)	12.9 (12.6)	11.6 (7.8)	5.1 (5.2)	6.2 (6.1)	6.5 (4.8)
CC 1/2	0.80	0.95	0.90	0.97	0.79	0.96	0.92
**Refinement**							
No. Reflections	18623	20095	35083	15736	13696	18299	22127
Rwork (Rfree) (%)	21.82 (24.78)	19.41 (24.35)	21.32 (26.18)	21.26 (26.61)	21.04 (26.72)	19.30 (24.95)	18.71 (24.50)
No. Complex in ASU	1	1	1	1	1	1	1
No. Atoms							
Protein	2320	2363	2327	2239	2244	2349	2336
DNA	1060	1058	1060	1060	1060	1063	1060
Ca2+	2	2	3	2	2	2	2
Water	39	197	109	15	35	90	102
B-factor	44.35	35.37	38.96	55.2	44.68	45.07	37.54
RMSD							
Bond lengths (Å)	0.003	0.003	0.015	0.002	0.005	0.004	0.011
Bond angles (°)	0.459	0.585	1.382	0.449	0.731	0.594	1.255
Ramachandran							
Preferred (%)	95.89	94.81	97.95	94.85	95.07	96.60	95.16
Allowed (%)	3.77	5.19	2.05	4.81	4.58	3.40	4.50
Outliers (%)	0.34	0.00	0.00	0.34	0.35	0.00	0.35

PDB ID	9Q7Z	9XYA	9PZH	9Q0H	9XY6	9XY1	11QF
	P456_CGG	P456_GGC_1	P456_GGC_2	P456_GGC_3	P456_TCC	P456_TGA	I-PnoMI
**Data Collection**							
Space group	C 1 2 1	P 21 21 21	P 21 21 21	P 21 21 21	P 21 21 21	P 21 21 21	I2
Cell dimensions							
a, b, c (Å)	327.80, 40.47, 78.24	39.56, 75.84, 166.80	39.31, 76.32, 168.32	39.28, 75.54, 167.23	43.19, 64.28, 167.83	38.67, 70.44, 165.94	76.94, 158.23, 76.96
α, β, γ (°)	90.0, 94.0, 90.0	90.0, 90.0, 90.0	90.0, 90.0, 90.0	90.0, 90.0, 90.0	90.0, 90.0, 90.0	90.0, 90.0, 90.0	90.0, 93.65, 90.0
Resolution (Å)	50.0-3.3 (3.42-3.30)	50.0-2.32 (2.40-2.32)	50.0-2.37 (2.45-2.37)	50.0-2.2 (2.28-2.20)	50.0 - 2.7 (2.80-2.70)	50.0-1.92 (1.99-1.92)	50.0-1.87 (1.91-1.87)
R_{Merge}	0.09 (0.15)	0.85 (0.11)	0.09 (0.30)	0.09 (0.59)	0.07 (0.39)	0.06 (0.67)	0.07 (0.75)
I/σ(I)	15.5 (6.5)	26.8 (2.2)	18.1 (5.1)	26.9 (3.4)	33.7 (4.7)	41.6 (3.7)	14.3 (2.1)
Completeness (%)	97.0 (79.8)	99.7 (99.2)	99.1 (93.9)	99.8 (98.4)	95.0 (73.8)	100.0 (100.0)	98.2 (90.3)
Redundancy	5.3 (3.4)	13.3 (10.0)	5.8 (4.7)	12.2 (9.6)	12.5 (9.4)	12.6 (12.1)	6.8 (5.8)
CC 1/2	0.99	0.88	0.92	0.94	0.96	0.90	0.99
**Refinement**							
No. Reflections	15020	22637	21481	25984	12656	35380	78699
Rwork (Rfree) (%)	24.47 (28.89)	21.27 (26.87)	17.85 (23.34)	19.84 (24.71)	23.90 (29.35)	20.48 (24.22)	18.31 (22.90)
No. Complex in ASU	2	1	1	1	1	1	2
No. Atoms							
Protein	3899	2320	2341	2283	2212	2273	4653
DNA	2120	1060	1059	1060	1058	1060	2123
Ca2+	2	2	3	6	3	8	4
Water	9	86	186	78	21	143	493
B-factor	99.13	49.23	34.66	45.69	49.11	34.19	33.18
RMSD							
Bond lengths (Å)	0.003	0.007	0.012	0.007	0.004	0.01	0.01
Bond angles (°)	0.58	0.894	1.266	0.913	0.589	1.126	1.138
Ramachandran							
Preferred (%)	94.89	94.88	95.90	95.56	94.74	96.91	98.49
Allowed (%)	4.23	4.10	4.10	4.10	4.91	2.75	1.51
Outliers (%)	0.88	1.02	0.00	0.34	0.35	0.34	0.00

Supplementary Table S1. Data collection and refinement statistics for the X-ray crystallographic structures used for this comparative analysis. All protein:DNA complexes were briefly incubated at room temperature, and crystals were grown using the hanging drop method as previously described in (29). Crystals were cryoprotected using 80% reservoir and 20% ethylene glycol or sucrose. Diffraction data was collected with an in-house Rigaku Micromax 007HF rotating anode generator and an RaxisIV++ imaging plate detector or remotely via the Advanced Light Source (ALS) synchrotron facility (Beamlines 5.0.1 or 5.0.2) at Lawrence Berkeley National Laboratory. All structures were solved with HKL2000 (47), PHASER (48), Phenix (49), COOT (50), and CCP4 (51), then submitted to the Protein Data Bank (RCSB.org) (52) using the PDB codes shown in this supplementary table and in **Table 1**.
